# Intravitreal NGF administration counteracts retina degeneration after permanent carotid artery occlusion in rat

**DOI:** 10.1186/1471-2202-10-52

**Published:** 2009-05-27

**Authors:** Sandra Sivilia, Alessandro Giuliani, Mercedes Fernández, Maria Elena Turba, Monica Forni, Alessandro Massella, Nadia De Sordi, Luciana Giardino, Laura Calzà

**Affiliations:** 1 DIMORFIPA, University of Bologna, Via Tolara di Sopra 50, 40064 Ozzano Emilia, Bologna, Italy; 2 BioPharmaNet-DIMORFIPA and National Institute of Biostructures and Biosystems (INBB), University of Bologna, Bologna, Italy

## Abstract

**Background:**

The neurotrophin nerve growth factor (NGF) is produced by different cell types in the anterior and posterior eye, exerting a neuroprotective role in the adult life. The visual system is highly sensitive to NGF and the retina and optic nerve provides suitable subjects for the study of central nervous system degeneration. The model of bilateral carotid occlusion (two-vessel occlusion, 2VO) is a well-established model for chronic brain hypoperfusion leading to brain capillary pathology, to retina and optic nerve degeneration. In order to study if a single intravitreal injection of NGF protects the retina and the optic nerve from degeneration during systemic circulatory diseases, we investigated morphological and molecular changes occurring in the retina and optic nerve of adult rats at different time-points (8, 30 and 75 days) after bilateral carotid occlusion.

**Results:**

We demonstrated that a single intravitreal injection of NGF (5 μg/3 μl performed 24 hours after 2VO ligation) has a long-lasting protective effect on retina and optic nerve degeneration. NGF counteracts retinal ganglion cells degeneration by early affecting Bax/Bcl-2 balance- and *c-jun- *expression (at 8 days after 2VO). A single intravitreal NGF injection regulates the demyelination/remyelination balance after ischemic injury in the optic nerve toward remyelination (at 75 days after 2VO), as indicated by the MBP expression regulation, thus preventing optic nerve atrophy and ganglion cells degeneration. At 8 days, NGF does not modify 2VO-induced alteration in VEFG and related receptors mRNA expression.

**Conclusion:**

The protective effect of exogenous NGF during this systemic circulatory disease seems to occur also by strengthening the effect of endogenous NGF, the synthesis of which is increased by vascular defect and also by the mechanical lesion associated with NGF or even vehicle intraocular delivery.

## Background

Nerve growth factor (NGF) is the best-known member of a family of neurotrophins (NTs) that regulate neuronal survival also during adult life [1]. Signals mediated by NGF are propagated by the high affinity receptor tyrosine kinase A (TrkA) and the low-affinity receptor p75, which is a member of the tumor necrosis factor receptor superfamily [2]. NGF plays key roles in the visual system development and function. Under normal conditions, NGF and TrkA are expressed in the anterior segment of the eye (iris, ciliary body, lens, cornea and conjunctiva), and NGF is released into the aqueous humour [3]. In the retina, NGF is produced and utilized by retinal ganglion cells (RGCs), bipolar neurons as well as glial cells, in a local paracrine/autocrine fashion. During visual system development, NGF, TrkA and p75, as well as other NTs and their related receptors, are highly expressed in numerous visual centers, from the retina to the visual cortex, where NGF influences neuritis outgrowth, survival and selective apoptosis [4,5]. A retrograde/anterograde transport along the axons of the RGCs and geniculate nucleus has been also reported [3]. Moreover, NGF exerts neurotrophic effects in vascular and capillary pathologies of the eye, as suggested in experimental diabetes [6] and after ischemia and reperfusion [7], such as in RGCs degeneration due to optic nerve section [8].

Models of retinal degeneration have been used to study changes in gene expression and protein synthesis preceding cell death [9]. In this study, we intended to verify if a single intravitreal injection of NGF protected the retina and optic nerve exposed to reduced blood supply. We tested this hypothesis in rats after bilateral occlusion of carotid arteries (two-vessel occlusion, 2VO). This is a well-established model for chronic brain hypoperfusion leading to brain capillary pathology, retina and optic nerve degeneration [9,8,10].

## Methods

### Animals and surgery

3-Month old male Sprague-Dawley rats (Charles River laboratories Italia, Calco, Lecco) were used for this study. The animals were housed in polypropylene cages, 4 animals per cage, under standard light/dark conditions (lights on 7:00, off 19:00) with food pellets and water *ad libitum*. Chronic cerebral hypoperfusion was induced by permanent bilateral occlusion of the common carotid arteries [8,9], with sham-operated animals serving as controls. Prior to surgery, the animals were anesthetized with ketamine hydrochloride (100 mg/kg ip). The common carotid arteries were exposed via a ventral midline incision, carefully separated from their sheaths and vagus nerves, and permanently doubly ligated with 5/0 silk suture approximately 8 to 10 mm below the origin of the external carotid artery. The incision was then sutured. The same procedure, except for artery ligation, was performed on the sham group. The mortally rate during or immediately after surgery was 12%. Twenty-four hours post surgery, intravitreal injection of saline (3 μl) or NGF solution (NGF, 5 μg/3 μl; NGF was generously supplied by Dr. Luigi Aloe, Institute for Neurobiology and Molecular Medicine, CNR, Roma, Italy; see Bocchini and Angeletti 1969 [11]) was performed in deeply anaesthetized rats, using a 25 μl Hamilton syringe with a 27-gauge needle through the pars plana at the temporal side of the eye [12]. The classical transcleral approach with the tip of a microsyringe tangentially inserted through a local sclerotomy was used [13]. Injections were performed slowly over a period of 2 minutes. In the case of intraocular bleeding, the animal was excluded from the study. As the estimated volume of the vitreous humor was about 60 μl [14], a 20-fold dilution of NGF was calculated. Therefore, the actual concentration of NGF delivered to the retina was 80 μg/ml.

Three different survival times (8, 30 and 75 days) after surgery were investigated for the purpose of morphological and morphometric studies, whereas mRNAs expression was investigated 8 days after ligation. In this experiment, we observed a 62% loss of pupillary reflex, thus of effectiveness of ligation. The loss of pupillary reflex could be both bilateral and unilateral [9]. For this reason, only the eye having lost the pupillary reflex was included in the study. Experimental groups were composed as shown in Table 1.

**Table 1:** Number of eyes included in study in the different experimental groups and at different post-surgical times.

**Days after ligation (or sham surgery)**	Sham-saline	Sham-NGF	2VO	2VO-saline	2VO-NGF
**8**	15	10	16	10	18

**30**	7	7	5	4	4
**75**	6	6	4	3	4

Only eyes showing lack of direct pupillary reflex during the first 24 h were included in the study (see text for further details)

All animal protocols described herein were carried out according to the European Community Council Directives of 24 November 1986 (86/609/EEC) and approved by our intramural committee and the Italian Health Ministry (123/2004-B), in compliance with the guidelines published in the *National Institute of Health Guidelines for the Care and Use of Laboratory Animals*.

### Pupillary reflex

We adopted pupillary reflex procedure described by Stevens et al. [9]. Each animal was first allowed to adapt to darkness for at least 5 minutes. One eye was then exposed to a beam of light from an otoscope to assess the direct reflex response. The otoscope was then immediately directed at the controlateral eye to assess the consensual response. Both eyes were then allowed to readapt to darkness for approximately 1 minute. This procedure was then repeated starting from the controlateral eye. Loss of pupillary reflex is defined as failure of the pupil to constrict after a 10-second of light exposure. Pupillary reflex was examined each day for a week after surgery, and at least once a week until animal sacrifice [9].

### Immunohistochemistry

All animals were deeply anesthetized with Ketamine (Ketalar, Parke Davis, Italy) 10 mg/kg body weight, i.p. (+ diazepam 2 mg/kg i.m.) and perfused through the ascending aorta, first with Tyrode-Ca^2+ ^free, pH 6.9 at 37°C (50 ml for adult male rats, 25 ml for young male rats), then with 4% paraformaldehyde in Sorensen phosphate buffer 0.1 M pH 7 at 37°C (50 ml and 25 ml for adult and young rats, respectively), finally with the same fixative, at an ice-cold temperature. During perfusion, animals were bathed in ice-cold water. The brains were then removed and immersed for 90 min in the same ice-cold fixative, before being rinsed for at least 48 h in 5% sucrose in 0.1 M phosphate buffer. Retinas were frozen in CO_2 _and 14 μm thick coronal sections were then obtained 300 μm from the optic disk using a cryostat (Kriostat 1750, Leica) and collected on gelatine coated slides. For immunofluorescence experiments, sections were first incubated in 0.1 M phosphate buffered saline (PBS) at room temperature for 10–30 min, followed by incubation at 4°C for 24 h in a humid atmosphere with the primary antibodies diluted in 0.3% PBS-Triton X-100, v/v (see Table 2 for the list of antisera used in this study). After rinsing in PBS for 20 min (2 × 10 min), the sections were incubated at 37°C for 30 min in a humid atmosphere with the secondary antisera conjugated with different fluorochromes: Cy™2- conjugated affinity-pure Donkey anti-Mouse IgG (H+L), Cy™2- conjugated affinity-pure Donkey anti-Goat IgG (H+L) and Rhodamine Red™-X-conjugated affinity-pure Donkey anti-Rabbit IgG (H+L) (all from Jackson Immunoresearch, West Grove, PA) diluted in PBS triton 0.3%. Sections were then rinsed in PBS (as above) and mounted in glycerol containing 1,4-phenylendiamine (0.1 g/l).

**Table 2:** Primary antisera and working conditions used in the study.

**ID**	**Antigens**	**Hosts**	**Suppliers**	**Dilution**
betaIII Tubulin	betaIII Tubulin	Rabbit	Santa Cruz	1:300
MBP	myelin basic protein	Rabbit	DAKO	1:100
Mcm-2	mini-chromosome maintenance	Goat	Santa Cruz	1:150
OX42	OX42	Mouse	Serotec	1:300
NG2	NG2 chondroitin sulfate Proteoglycan	Mouse	Chemicon	1:100
Laminin	Laminin	Rabbit	Sigma	1:25

### Morphometric analysis

Histological and immunohistological images were captured on a Nikon Eclipse E600 microscope equipped with a Nikon color digital camera DXm 1200F. Confocal Laser scanning Microscopy (Olympus IX80 equipped with FluoView 500 software and 3 laser beam Ar 488, G-HeNe 543, R-HeNe 633 μm) was also used. Measurements were performed using the Image Pro-Plus software (Media Cybernetics, MD, USA). The intra-orbital portion of the optic nerve was analyzed. The diameter of the optic nerve was measured in 5 sections every 150 μm in the stamp (1 mm) proximal to the optic bulb. The immunoreactive area was calculated as a fraction (percentage of immunoreactive area) of the entire transversal section of the optic nerve. The thickness of the retina layers was calculated by dividing each retina, as sampled with coronal sections collected at 300 μm rostral to the optic disk, into 4 quadrants. Values are the mean ± SEM in all quadrants. The number of ganglion cells was estimated along the entire perimeter of the retina by counting nuclear profiles.

### RNA isolation and reverse transcription

Total RNA (total RNA isolation kit, Roche Molecular Biochemicals) was obtained from the inner tunica of the eye including retina and subjected to DNase treatment (1 U/μl, Fermentas, Life Sciences, Italy) in the presence of 4 U/μl RNase inhibitor (4 U/μl, Fermentas), incubating at 37°C for 30 min. First strand cDNAs were obtained after incubation with 200 U of the M-Moloney murine leukaemia virus (M-MuLV) reverse transcriptase enzyme (Fermentas) in a mix reaction composed of 1× First Strand Buffer, 1 mM of each d(NTP)s (Fermentas) and 5 μM of the p(dN)_6 _random primers (Roche Molecular Biochemicals), at 37°C for 50 min, followed by denaturation step heating at 70°C for 15 min.

### Relative Quantitative real-time PCR

Relative Quantitative real-time PCR reactions were performed in the Mx3005*P*™ real-time PCR system (Stratagene, CA, USA) using SYBR-green I dye. Rat primers for BAX and Bcl-2, pro- and anti-apoptotic genes, respectively, NGF and its high and low affinity receptors, trk-A and p75, respectively, VEGF-A and its receptors FLT-1 and FLK-1 and GAPDH as housekeeping gene, real-time PCR were designed for SYBR-green chemistry using Beacon Designer 4.0 software (Premier Biosoft International, Palo Alto, CA, USA). The sequences of the designed primers as well as the accession number in the gene bank database are described in Table 3. For the amplification of NGF, trk-A, p75, VEGF-A, FLT-1 and GAPDH, the reactions were performed in a final volume of 25 μl consisting of 1× master mix (Brilliant SYBR-green QPCR master mix, Stratagene), 16 nM reference dye Rox (Stratagene) and 0.4 μM forward and reverse primers. Two step PCR reactions (40 cycles of denaturation 95°C for 15 sec, annealing/extension 60°C for 30 sec) were performed after enzyme activation (95°C, 10 min); at the end of PCR reaction the melting curve of amplified products was performed following this temperature/time scheme: heating from 55°C to 95°C with a temperature increase of 0.5°C/sec. In the case of FLK-1, the amplification program consisted of 40 cycles of denaturation- 95°C for 15 sec, annealing- 58°C for 20 sec, extension- 72°C for 20 sec); the melting curve of amplified products was performed this time starting from 53°C and following the same temperature/time scheme.

**Table 3:** Sequences of primers used for real-time semiquantitative PCR reactions

*Primer*	Acc. N°	Sequence (5'-3')	Amplified product (bp)
Bax	AF235993	5'-tgctacagggtttcatccag-3'	135
		5'-ccagttcatcgccaattcg-3'	
Bcl-2	NM_031535	5'-tggaaagcgtagacaaggagatgc-3'	88
		5'-caaggctctaggtggtcattcagg-3'	
*c-jun*	X17163	5'- aacgaccttctacgacgatg-3'	140
		5'-ggtgcggcttcagattgc-3'	
NGF	M36589	5'- acctcttcggacactctgg-3'	165
		5'- cgtggctgtggtcttatctc-3'	
P75	NM_012610	5'- agtggcatctctgtggac-3'	130
		5'- ctacctcctcacgcttgg-3'	
TrkA	NM_021589	5'- aagccgtggaacagcatc-3'	91
		5'- agcacagagccgttgaag-3'	
VEGF	AY702972	5'-accctggctttactgctgtac-3'	103
		5'- cgtccatgaacttcaccacttc-3'	
FLT-1	NM_019306	5'- tgtcctcaactgcaccgtcac-3'	100
		5'- ccgctgcctgatagatgctctc-3'	
FLK-1	NM_013062	5'- tggcaaatacaacccttcag-3'	108
		5'- cacactcagtcaccaacac-3'	
GAPDH	M17701	5'-ggcaagttcaatggcacagtcaag-3'	125
		5'-acatactcagcaccagcatcacc-3'	

The relative mRNA level of studied genes was calculated on the basis of the threshold cycle (C_T_) values obtained for each sample. The housekeeping gene GAPDH was used to normalize the amount of retro-transcribed mRNA used for PCR reactions. The relative changes in mRNA expression were examined using the 2^-ΔΔCt ^method [15]. Before processing, the samples corresponding to the different groups of animals, the efficiencies of Bax, Bcl-2, *c-jun*, NGF, p75, TrkA, VEGF-A, Flt-1, Flk-1 and GAPDH real-time PCR reactions were calculated by amplifying cDNA dilution series in the presence of the corresponding primers in the conditions described above. Threshold cycle (C_T_) values for each pair of primers were used by the corresponding real-time QPCR instrument software (Stratagene, CA, USA or BioRad) to calculate standard curve slopes and efficiencies. For all pairs of primers used herein we obtained slope and efficiency values that allowed us to calculate the relative expression of all genes studied using the formula X = 2^-ΔΔCt^, where ΔΔCt = average ΔCt (target gene in a given experimental animal group) - average ΔCt (target gene in the corresponding control animal group); ΔCt (target gene of a experimental animal group) = Ct (target gene) - Ct (GAPDH), for all animals within this experimental animal group; ΔCt (target gene of a control animal group) = Ct (target gene) - Ct (GAPDH), for all animals within this control animal group.

The specificity of real-time PCR reactions was evidenced by the melting curve, with a unique peak being obtained at the corresponding melting temperature (Tm) and by agarose gel electrophoresis of random PCR products for each pair of primers used, obtaining a single band of the expected size.

### Statistical analyses

Graph preparation and statistical analyses were performed using GraphPad Prism 4.0 software. For real-time PCR experiments from 4 to 6 animals per group were used and experiments were performed in triplicate. Data are presented as mean values ± S.E.M. Statistical analyses: 1 way ANOVA and post-hoc Tukey's test were used to compare different experimental groups or else Student's t test, when only two groups were compared (see legends to the figures for further details).

## Results

### Animals

The bilateral permanent occlusion of the carotid arteries induces the loss of the pupillary reflex within 24 h after ligation in 62% of the animals included in the study. This is a permanent deficit, as defined by observation at 8, 30 and 75 days after 2VO. In order to make the experimental groups more homogeneous, only animals with direct bilateral or unilateral pupillary reflex loss within this post-surgical time were included in the study. In the case of unilateral lesions, only the lesioned eye was considered. Table 1 reports the number of eyes included in the study in the different experimental groups. NGF intravitreal administration performed 24 h after 2VO ligation failed to induce any functional recovery, as evaluated by pupillary reflex.

### Retinal and optic nerve degeneration in 2VO

**Figure 1:**
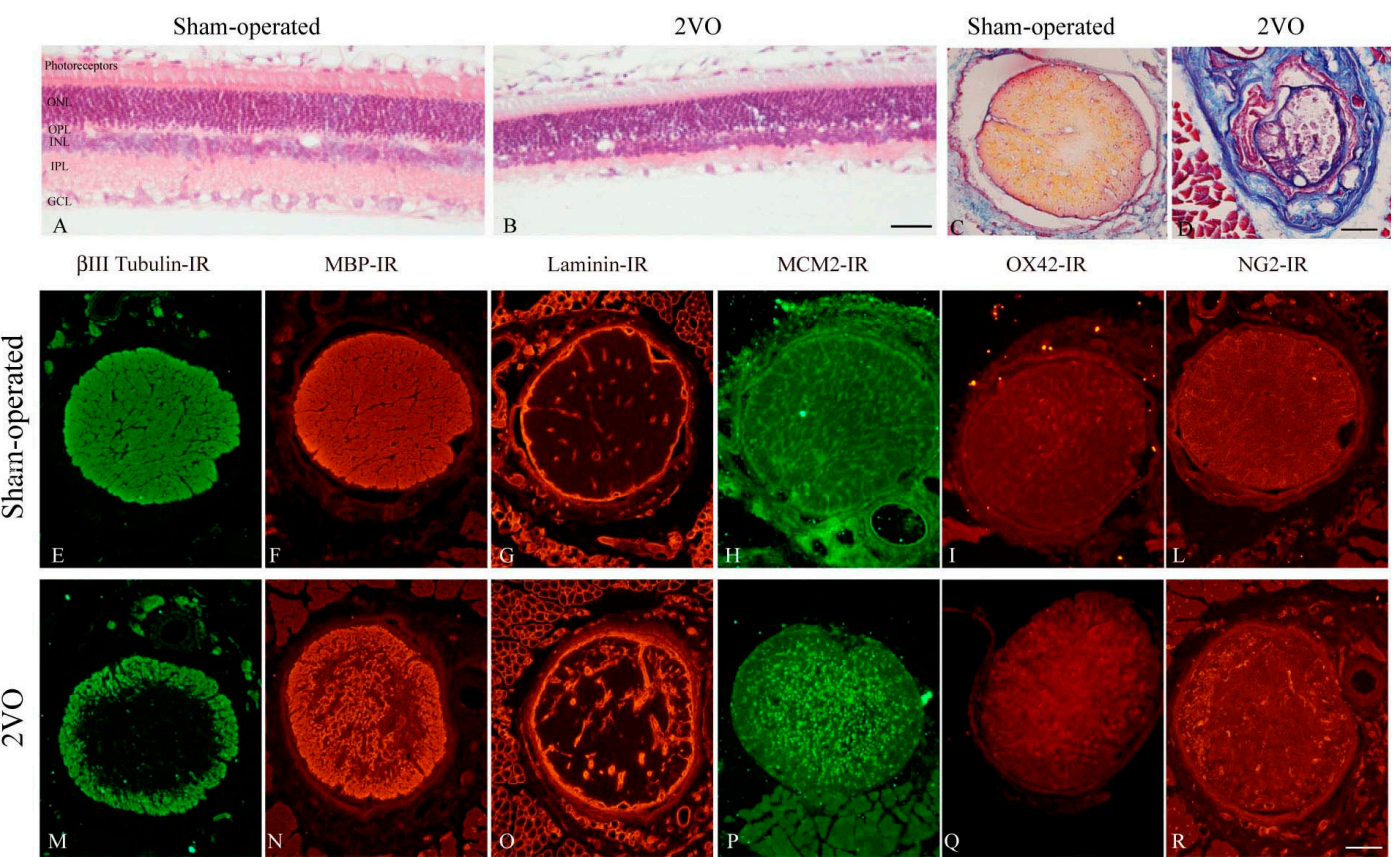
**A, B: Histological staining (Hematoxylin-Eosin) of the retina of sham-operated (A) and 2VO ligated 8 days after surgery (B)**. C, D: Histological staining (Masson Trichromic staining) of the optic nerve of sham (C) and 2VO animals 8 days after surgery (D). E-R: Optic nerve staining of sham- (E-L) and 2VO- operated animals 8 days after surgery (M-R) with the markers listed in the figure. Images are obtained by conventional fluorescence microscopy. Bars: 100 μm (A, B); 200 μm (C-R). *Abbreviations: ONL*: outer nuclear layer; *OPL*: outer plexiform layer; *INL*: inner nuclear layer; *IPL*: inner plexiform layer;*GCL*: ganglion cell layer.

**Figure 2:**
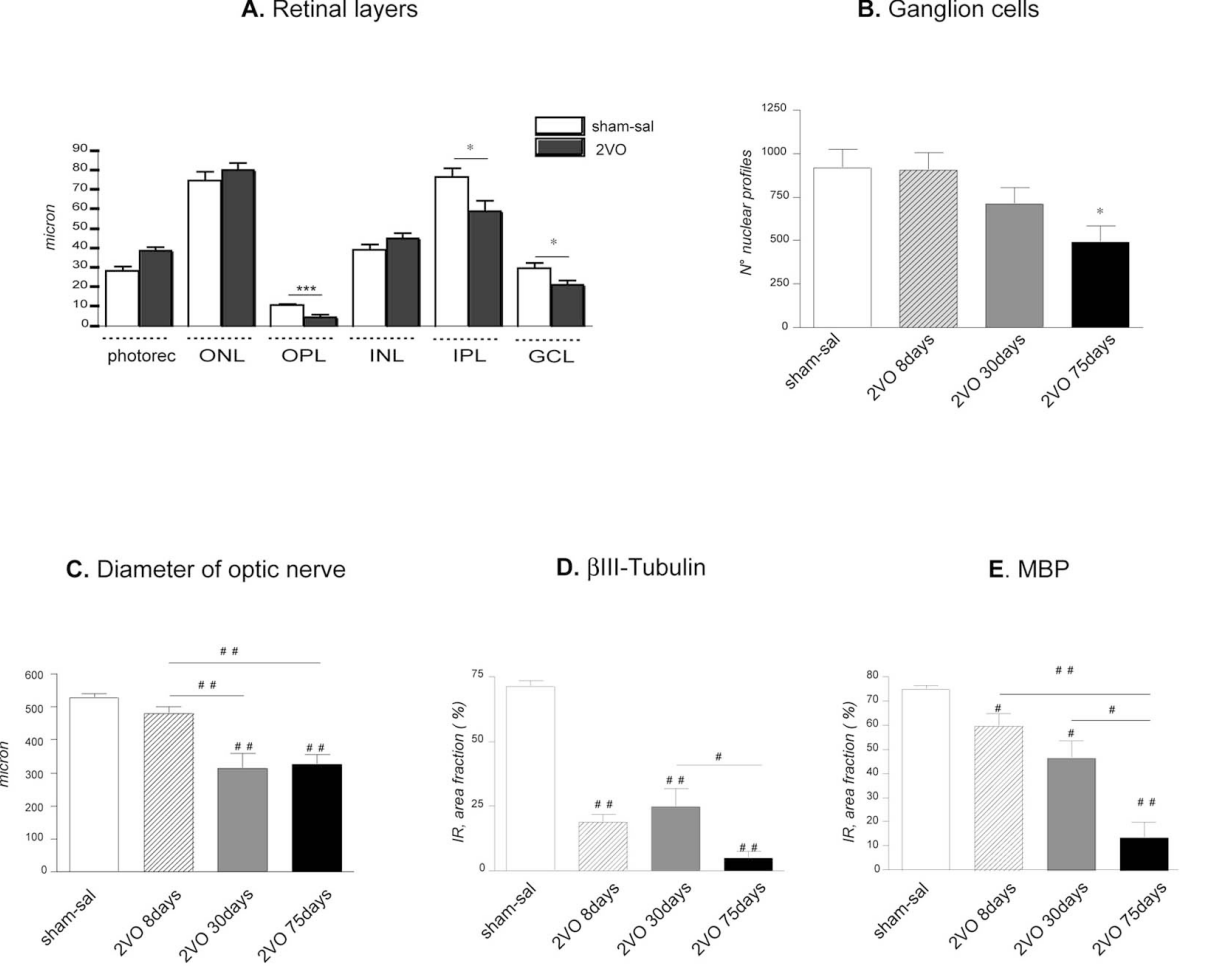
**Morphometric analysis of the retina and optic nerve in the experimental groups**. In the retina, the OPL layer almost disappears in 2VO animal 8 days after surgery (A; Student-t test: ***p < 0.0001). A reduction in the number of ganglion cells 75 days after surgery was also found (B, Student's *t *test: *p < 0.05). The atrophic effect of ligation is shown as reduction of the optic nerve diameter (C; one-way ANOVA and post hoc Tukey's test: ^##^p < 0.001). The semiquantitative analysis of relative immunoreactive area for the betaIII-Tubulin (D) shows a strong reduction at 8, 30 and 75 days after ligation (one-way ANOVA and post hoc Tukey's test: ^#^p < 0.05, ^##^p < 0.001), such as the analysis of MBP (E, one-way ANOVA and post hoc Tukey's test: ^#^p < 0.05; ^##^p < 0.001). *Abbreviations: ONL*: outer nuclear layer; *OPL*: outer plexiform layer; *INL*: inner nuclear layer; *IPL*: inner plexiform layer;*GCL*: ganglion cell layer.

Permanent bilateral occlusion of the common carotid arteries causes a degeneration of the retina and optic nerve, as shown by the histopathology of 2VO *versus *sham-operated rat (control) (Fig. 1). In 2VO animals, the OPL of the retina almost completely disappears 8 days after ligation, and a reduction of IPL and GCL is also observed (Fig. 2A). The estimated number of ganglion cells in the whole perimeter of the retina decreases 75 days after 2VO ligation (Fig. 2B). Atrophy of the optic nerve, as assessed by nerve diameter, is observed 30 and 75 days after surgery (Fig. 2C). In order to monitor anatomical and molecular changes induced by the vascular defect in the optic nerve, we analysed beta-tubulin as marker for microtubules, MBP as marker for myelin, laminin as marker for vascular bed and scar formation, MCM2 as marker for proliferating cells, OX42 as marker for activated microglia and NG2 as marker for oligodendrocyte precursor cells (OPCs). Sample images of the indicated immunohistochemical staining in control sham operated and 2VO ligated animals at 8 days after surgery are shown in Fig 1. We observed a rapid degeneration of axonal integrity starting from the central portion of the nerve, as indicated by the decrease in beta-tubulin and MBP immunostaining, which is linked to an increase in the vascular bed, as depicted by laminin-IR. We also found an impressive increase in cell proliferation, which might be referred to glial cells including microglia (OX42-IR) and oligodendrocyte precursor cells (OPCs) (NG2-IR). We then used beta-tubulin and MBP as markers for axon analysis, and the graphs in Fig. 2 report the evaluation of immunoreactive areas for these antigens, showing a rather rapid microtubule disaggregation leading to an almost complete depletion of beta-tubulin in the atrophic nerve (75 days) (Fig. 2D). Immunostaining for the myelin sheet associated with MBP protein decreases more slowly, again starting from the core of the nerve (Fig. 2E).

### NGF administration and retinal and optic nerve degeneration

After characterization of the time-course of the retinal and optic nerve pathology after 2VO ligation, we explored the possible protective effect of a single NGF intravitreal injection. We chose to inject NGF 24 hours after 2VO ligation for two main reasons. The first was technical in nature: pupillary reflex loss, which was established as an inclusion criteria for the study could develop until 24 hours after ligation [9]. The second was related to the specific aim of the study: we intended to evaluate if NGF is able to interfere with a degeneration process already under way.

**Figure 3:**
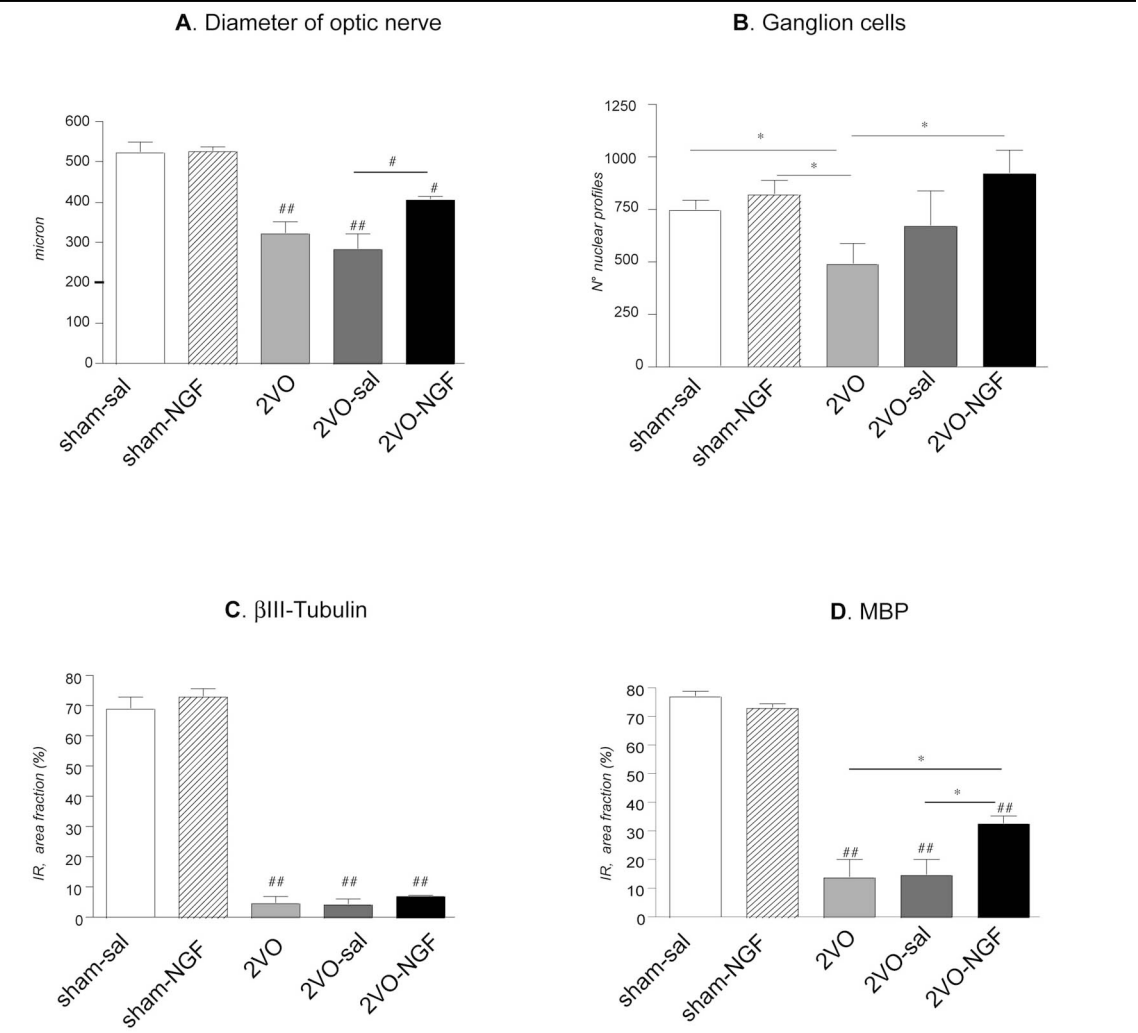
**Morphometric analysis of NGF effect on optic nerve diameter (A), ganglion cells (B), betaIII-Tubulin (C) and MBP (D) immunostaining**. NGF administration partially prevents 2VO-induced optic nerve atrophy (A) and ganglion cells degeneration at 75 days after surgery. NGF also induces a partially recovery of MBP- immunoreactivity (D), whereas betaIII-tubuline immunostaining is not modified (C). Statistical analysis: one-way ANOVA and post hoc Tukey's test: ^#^p < 0.05; ^##^p < 0.01 and Student-t test: *p < 0.05.).

In this part of the study, we compared 5 experimental groups: sham operated animals, sham operated animals which were intravitreally injected with saline or NGF; 2VO animals which were intravitrealy injected with saline or NGF; and 2VO animals with intact eyes. In order to allow NGF diffusion in the humor vitreo, we used an injection volume of 3 μl, which displayed regular reproducibility with minimum loss of injected solution [13] and induced an increase in vitreous humor volume of about 5% [14]. We evaluated the NGF effect on optic nerve and retina degeneration by estimating the number of ganglion cells 75 days after 2VO legation, and by measuring the optic nerve diameter, and the beta-tubulin- and MBP-immunostained area fraction over the optic nerve transverse section. The data are presented in Fig 3. A significant preservation of optic nerve diameter and ganglionic cells with nuclear profile is observed in 2VO-NGF- compared to 2VO-saline-treated rats. This effect is linked to a positive effect on MBP-, but not beta-tubulin immunostaining.

**Figure 4:**
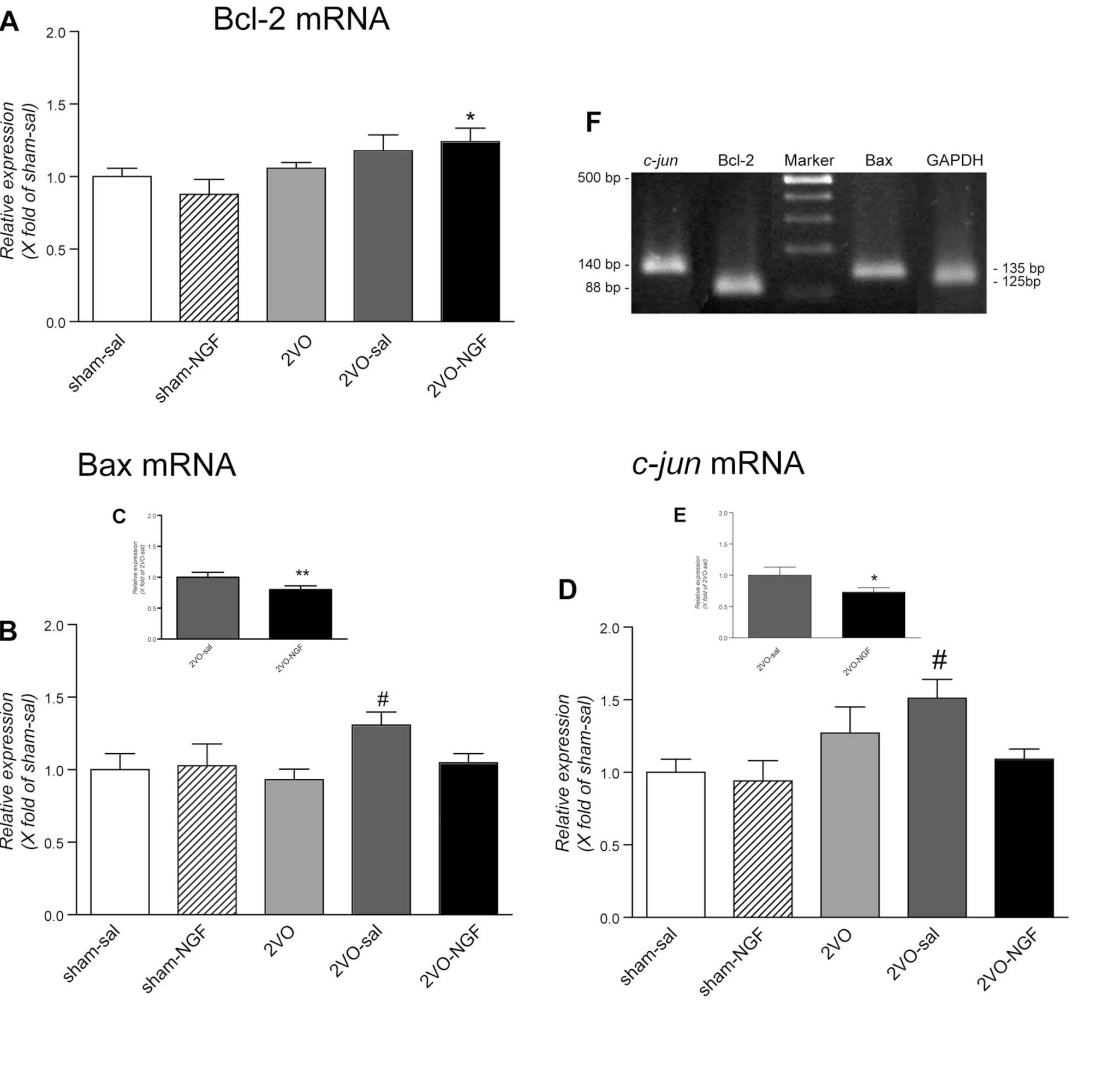
**Real time PCR of Bax, Bcl-2 and *c-jun *genes**. The induction of hypoperfusion induces an increase in the pro-apoptotic gene Bax mRNA level (2VO-saline *versus *sham-saline) in the retina (B) which then decreases after NGF treatment. The relative expression of the anti-apoptotic gene Bcl-2 conversely increases in the 2VO-NGF group compared with the sham-saline group of animals (A). The vascular lesion also causes the induction of *c-jun *mRNA expression (2VO-saline *versus *sham-saline) (D), which is counteracted by NGF intravitreal injection (E). Experiments were performed in triplicate. Results are presented as mean values ± SEM. Statistical analyses performed: one-way ANOVA with multiple comparison post hoc Tukey's test (^#^p < 0.05) and Student's *t *test (*p < 0.05; **p < 0.01).

In order to explore whether the protective effect of NGF on retinal ganglion cells degeneration induced by ischemic insult is related to an anti-apoptotic effect of NGF, we investigated the expression profile of genes involved in apoptosis, e.g. Bax/Bcl-2 and *c-jun *by real-time PCR, 8 days after ligation (Fig. 4). Data are expressed as relative gene expression in folds of control group (sham-saline). In the 2VO group, the expression of pro-apoptotic gene Bax increases in saline-injected, but not in NGF-injected animals (Fig. 4C), while an increase in the anti-apoptotic gene Bcl-2 mRNA is observed in this group (Fig. 4A). Interestingly, Bax up-regulation is observed in animals having undergone double surgical manipulation, e.g. vascular ligation and intravitreal injection (2VO-saline compared with sham-saline) (Fig. 4B). The same effect by both vascular and mechanical lesions was also observed in the expression of the immediately early gene (IEG) *c-jun *mRNA at the same time-point (Fig. 4D). NGF counteracted the increase in the expression of *c-jun *mRNA in 2VO group of animals (2VO-NGF compared with 2VO-saline) (Fig. 4E). The expression profile of *c-jun *is the same as that observed for Bax.

**Figure 5:**
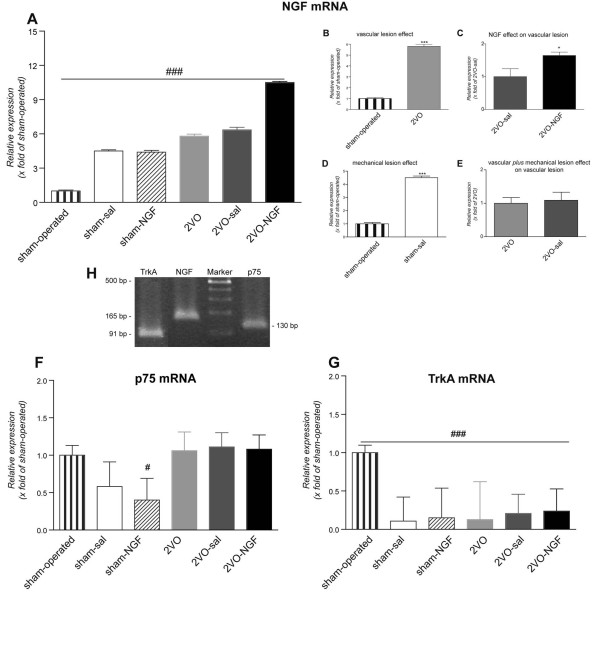
**Relative mRNA expression of NGF and its receptors p75 and TrkA in the retina at 8 days after vessel ligation**. NGF mRNA increased in all the experimental groups when compared with the sham-operated group of animals (A) and also in the 2VO-NGF group when compared with 2VO-saline (NGF effect on vascular lesion) (C). The opposite effect was found for the high affinity NGF receptor, TrKA, with a decrease observed in the mRNA level in all the experimental groups compared with the sham-operated group of animals (G). The mRNA level of the low affinity NGF receptor p75 is not significantly altered in the experimental groups studied except in the case of NGF injected sham group of animals (F). Results are presented as mean values ± SEM of experiments performed in duplicate. Statistical analysis was performed by one-way ANOVA and post hoc Tukey's multiple comparison test (^#^p < 0.05; ^###^p < 0.001) or Student's *t *test (*p < 0.05).

Since ocular tissues actively synthesize NGF, we then analyzed endogenous NGF and related receptor mRNA expression in the inner tunica including the retina. Since intraocular production of NGF is strongly regulated by lesions, such as intravitreal injection and vascular defects, we utilized as reference group sham-operated animals in this group of experiments, in which non intravitreal injections were performed. In all experimental groups we found an increase in NGF mRNA expression (Fig 5A). In particular, both mechanical and vascular lesions promote the expression of NGF mRNA (Fig. 5B,D), and double lesions (intravitreal injection plus vascular defect) further increase it (Fig. 5E). When NGF was administered in 2VO-animals, the local production of NGF mRNA increased more than 10 fold compared with sham operated animals but the increase remained statistically significant compared with 2VO saline-injected animals.

The expression of TrkA mRNA is strongly downregulated by injection and vascular lesions, whereas vascular lesions increase p75 expression and this effect is not regulated by NGF administration (Fig. 5G, F).

**Figure 6:**
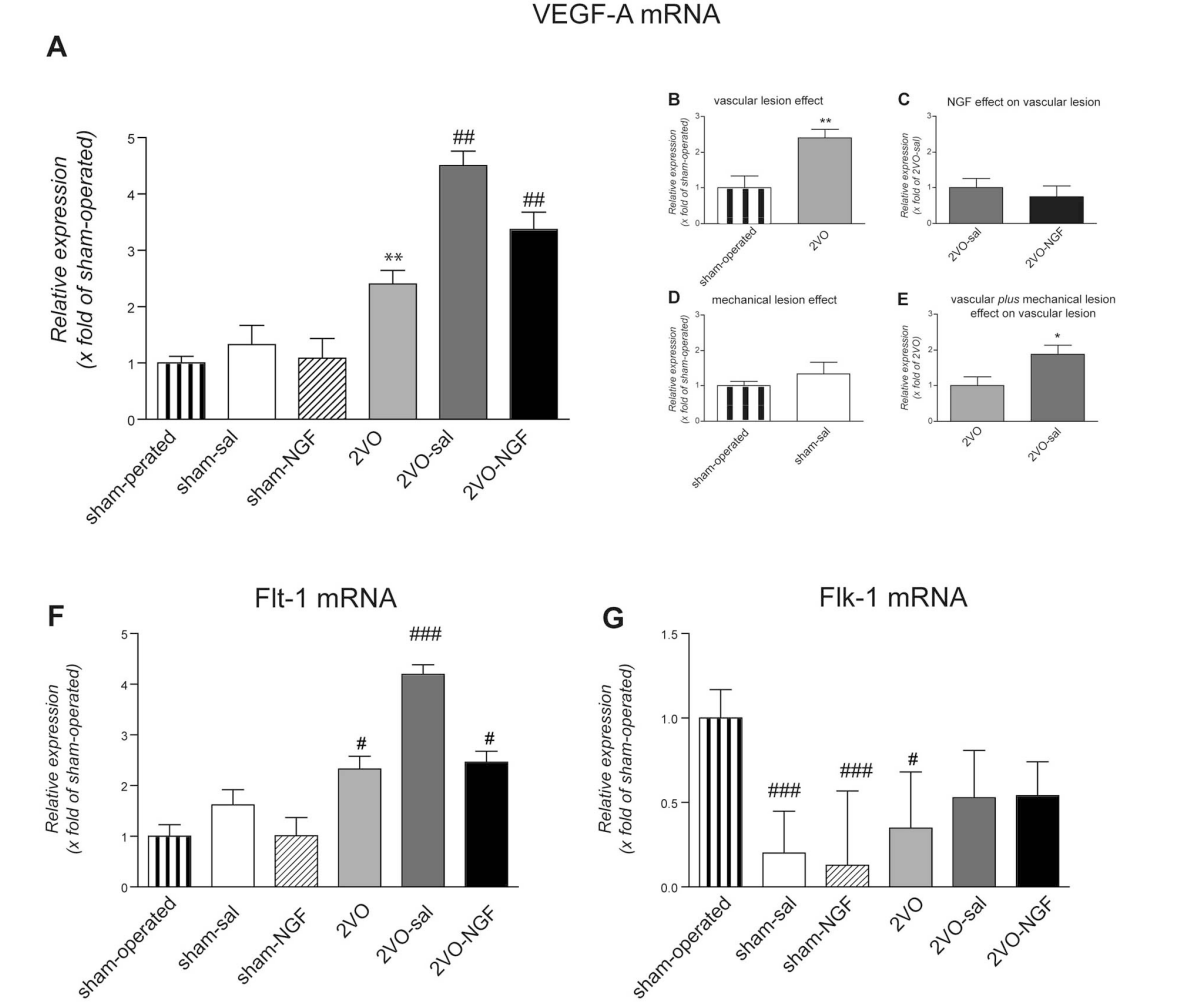
**Relative mRNA expression of VEGF-A, Flt-1 and Flk-1 in the retina at 8 days after vessel ligation**. The relative expression of VEGF (A) and its Flt-1 receptor (F) is increased in the 2VO groups with respect to the sham group of animals while the relative expression of Flk-1 decreases in 2VO groups compared with sham-group (G). NGF injection reduced the damage-induced VEGF mRNA expression. Results were considered statistically significant when one-way ANOVA and post hoc Tukey's test, ^# ^p < 0.01, ^### ^p < 0.001; Student's *t *test: *p < 0.05.

The mRNA expression of the angiogenic factor VEGF increases in the retina of 2VO compared to sham-operated animals 8 days after ligation. In particular vascular lesions promote expression of VEGF mRNA (Fig. 6B) whereas mechanical lesion-induced increase is not statistically significant (Fig. 6D). Double lesion (intravitreal injection plus vascular defect) further increases VEGF mRNA expression level (Fig. 6E). When NGF was administered in 2VO-animals this synergic effect was not detected (Fig. 6C).

The expression of FLK-1 mRNA is strongly down-regulated by injection and vascular lesion (Fig. 6G). Vascular lesion increases FLT-1 mRNA expression and double lesions further increase it; this effect is reversed by NGF administration (Fig. 6F).

## Discussion

Permanent bilateral occlusion of the common carotid arteries is a model of chronic cerebral hypoperfusion leading to a progressive loss of hippocampal neurons, cholinergic dysfunction and cognitive impairment. It also affects the retina and optic nerve, thus providing a useful model for investigating the mechanisms of and therapy for the retinal degeneration associated with vascular defects [9]. In this study we demonstrated that a single intravitreal injection of NGF has a protective effect on retina and optic nerve degeneration involving the early regulation of pro- and anti-apoptotic gene expression. Exogenous NGF seems to strengthen the effect of endogenous NGF, the synthesis of which is increased by vascular defect and also by the mechanical lesion associated with NGF or even vehicle intraocular delivery.

### Animals and model characterization

Permanent bilateral ligation of the carotid arteries causes rapid optic nerve lesion and loss of the pupillary reflex, followed by retrograde dysfunction and apoptosis of RGCs [9,16]. Only a subset of 2VO rats (60%) loses the pupillary reflex and suffers visual system pathology. This is probably due to the high individual variability of the Willis circle structure, which allows reverse perfusion of the retina through internal carotid and thence pterygopalatine arteries that branches from the internal carotid and supplies blood to the optic nerve [17,8]. Rats without this characteristic may suffer more severe ischemia and lose their pupillary reflex [8,17,9]. We included only affected eyes in our study. Moreover, we analyzed the retina and the intra-orbital portion of the optic nerve, that is, areas both supplied by the central artery of the retina, which derives from of the internal carotid through the ophthalmic artery [18,19].

According to the hallmarks of the model [20,10] retinal degeneration is characterized by a severe reduction of retinal layer thickness, particularly in the OPL and IPL layers, already 8 days after 2VO, whereas the number of RGC nuclear profiles decreases 75 days after ligation. The marked disaggregation of microtubules and myelin disintegration in the optic nerve is accompanied by microglial activation [16,21,22]. Degeneration of the optic nerve fibers accompanies the progressive death of RGCs.

In our experimental conditions, two pathogenic events might influence degeneration of RGCs: the first one is direct retina and optic nerve injury caused by a vascular defect (hypoperfusion); the second one is eye puncture and/or injection. In fact, according to Sobrado-Calvo *et al*. [22], eye puncture itself or the increase in intraocular pressure due to injection of substances can induce eye damage. An increase in vitreous humor volume of about 5% [14] induced by an injection volume of 3 μl could contribute to retinal degeneration. Optic nerve fibers could be compressed by high intraocular pressure, producing a disruption of retrograde transport of neurotrophins from the superior colliculus, along the optic nerve, to the RGC soma [23,24,3,26]. We actually found an increase in NGF mRNA expression 8 days after 2VO ligation. At this time, a rapid and transient activation of OPCs (NG2-IR cells) and microglial cells is observed in the optic nerve, probably reflecting inflammation [27].

### NGF protects retina and optic nerve from vascular lesion

NGF is a potent stimulant of the trophism and wound healing process in the eye [28] and basically all eye structures are able to produce NGF and even to up-regulate NGF synthesis in the case of lesion [3]. In our study we found that NGF-injection, but also vehicle injection, strongly stimulates NGF synthesis in the retina, thus confirming that endogenous NGF plays a critical role in retina and optic nerve protection, also in vascular lesion. Moreover, this increase, observed after 8 days, is further stimulated by hypoperfusion and even more by exogenous NGF. NGF injected intravitrealy has a great capability to diffuse and to reach the optic nerve itself [29], as also indicated by the brain diffusion of NGF corneal drops [30]. A single NGF injection induced the regulation of Bax/Bcl-2 gene expression balance and also the expression of the IEG *c-jun *in the retina at the earlier time studied (8 days after 2VO ligation), and the effect of this single dose of NGF was long lasting, as indicated by preservation of the optic nerve diameter and myelination and by RGC profile counting.

There are several possible stages at which NGF could interfere with retinal and optic nerve degeneration and different cellular targets, including: direct effect on RGC degeneration, indirect effect on RGC retrograde degeneration through axon protection, myelin repair promotion, neoangiogenesis and vasculogenesis promotion.

NGF counteracts RGC degeneration by affecting Bax/Bcl2 balance- and *c-jun*- expression, suggesting a direct effect of NGF on retinal cells. This effect is early and long-lasting. Axotomy and glaucoma cause apoptosis of RGCs, as has been demonstrated by expression studies of caspases, Bcl-2/Bax and c-jun [31-33]. RGC death begins 3 days after axonal lesion and is preceded by an early activation of Bax [32]. Moreover, vascular injury also increases the expression of stress-related proteins, including death-shock proteins as c-fos and DNA nuclear fragmentation in the retina [34]. In these conditions, neurotrophins support survival of injured RGCs [12,29,22]. A similar anti-apoptotic effect of exogenous NGF has also been described in experimental retinal detachment [35,29].

NGF could also regulate demyelination/remyelination balance after ischemic injury in the optic nerve, as indicated by the MBP expression regulation. At low concentrations, NGF enhances survival of the oligodendrocyte, the myelinating and remyelinating cell of the optic nerve [36], favouring fiber regeneration and proliferation [37] while at high concentrations it elicits apoptosis of mature cortical oligodendrocytes in vitro [38]. NGF stimulates oligodendrocyte differentiation, as defined by the elevation of myelin basic protein (MBP) in postmitotic cells [39]. NGF also regulates axonal signals that control myelination, promoting myelination by Schwann cells but reducing myelination by oligodendrocytes [40,41]. Finally, hypomyelination of the optic nerve has been observed in mice lacking BDNF [42,25]. In sum, the picture of the possible involvement of neurotrophins and related receptors in myelination is still partial and in some instances, controversial [43].

NGF has also been indicated as a novel angiogenic molecule that may contribute to the maintenance, survival, and function of endothelial cells by autocrine and/or paracrine mechanisms [44,45] and the reciprocal regulation between VEGF and NGF might also result in neuroprotection [46]. As expected, VEGF and Flt-1 mRNA expression is up-regulated in 2VO animals, whereas NGF administration is ineffective in this lesion-induced regulation. However, time course experiments are to be recommended in order to explore possible cross talk between NGF and VEGF signalling.

We also explored NGF high (TrkA) and low (p75NTR) affinity receptor mRNA expression regulation, observing a decrease in TrkA expression in all groups compared to the sham-operated group (the only one without any eye damage), and a slight decrease in p75NTR mRNA expression in NGF-injected eyes. No up-regulation of p75NTR mRNA expression was observed in 2VO animals. We studied NGF receptor expression at only one time-point, and might thus have missed earlier and late expression regulation. In fact, p75 NTR mRNA expression increases in retina during experimental glaucoma starting at 20 days, corresponding to increased Bcl-2/Bax ratio and apoptotic cell death [47].

Indeed, the contribution of the NGF/TrkA/p75NTRp75 signalling pathways to cellular activities is complex and still largely unknown [48,2,49]. NGF also plays a crucial role also during nerve injury and in the regulation of immune and inflammatory responses [49]. According to this, the expression regulation of NGF receptors is a distant reflexion of possible cellular action by NGF. P75NTR is normally scarcely expressed in the retina, and its expression is differentially regulated also according to the chronology of the lesion. For example, p75 expression increases 3–5 days after ischemia, and then decreases further [50]. Conversely, ocular hypertension induces a late increase in p75 expression in the retina, i.e. after 28 days [25] and apoptosis is associated with a consistent p75 expression [3,51].

## Conclusion

In this study we showed that a single intra-vitreal NGF injection protects retina and optic nerve from degeneration due to vascular injury. This effect is also mediated by an increased synthesis of endogenous NGF due to the mechanical lesion associated with intraocular delivery.

## Authors' contributions

SS executed surgery, the experimental part of immunohistochemistry and image analysis. AG: executed surgery. MF participated the Real Time PCR. MT and MF participated the VEGF and receptor mRNAs analysis. AM contributed on the execution of the Real Time PCR. NDS partecipated the histology. LG: partecipated in the design of the study design and in the manuscript preparation. LC conceived of the study and participated in its design, performed the statistical analysis, and coordinated and helped to the final version of the manuscript.
